# A partly-contacted epitaxial lateral overgrowth method applied to GaN material

**DOI:** 10.1038/srep23842

**Published:** 2016-04-01

**Authors:** Ming Xiao, Jincheng Zhang, Xiaoling Duan, Hengsheng Shan, Ting Yu, Jing Ning, Yue Hao

**Affiliations:** 1 School of Microelectronics, Xidian University, Xi’an, 710071, China

## Abstract

We have discussed a new crystal epitaxial lateral overgrowth (ELO) method, partly-contacted ELO (PC-ELO) method, of which the overgrowth layer partly-contacts with underlying seed layer. The passage also illustrates special mask structures with and without lithography and provides three essential conditions to achieve the PC-ELO method. What is remarkable in PC-ELO method is that the tilt angle of overgrowth stripes could be eliminated by contacting with seed layer. Moreover, we report an improved monolayer microsphere mask method without lithography of PC-ELO method, which was used to grow GaN. From the results of scanning electron microscopy, cathodoluminescence, x-ray diffraction (XRD), transmission electron microscopy, and atomic force microscope (AFM), overgrowth layer shows no tilt angle relative to the seed layer and high quality coalescence front (with average linear dislocation density <6.4 × 10^3^ cm^−1^). Wing stripes peak splitting of the XRD rocking curve due to tilt is no longer detectable. After coalescence, surface steps of AFM show rare discontinuities due to the low misorientation of the overgrowth regions.

Epitaxial lateral overgrowth (ELO) has been shown to significantly reduce extended defect densities in heteroepitaxy GaN film^1^,^2^,^3^,^4^,^5^. For conventional ELO, difficulties remain in controlling the structural quality of overgrown material during coalescence between neighboring features (e.g., stripes)^6^. It has been observed that the “wing” (overgrown GaN) exhibits crystal orientation tilts (wing tilts) away from the “window” (seed) regions, in an azimuth perpendicular to the stripe direction^3^. Wing tilt is detrimental to the coalescence of neighboring stripes, since the coalescence front takes the form of a tilt boundary with twice the magnitude of the average wing tilt. Since the crystal planes in the wings have distinctly different orientations compared to their counterparts in the window regions, these tilts deteriorate the coalescence fronts and lead to high linear threading dislocation density (TDD) at the coalescence regions^7^. The measurement of wing tilt is made with the scattering plane (defined by incident and diffracted wave vectors) perpendicular to the nucleation stripe direction^6^. In this orientation, an ω rocking curve about the window GaN 0002 peak reveals wing tilt as a splitting of peaks.

Besides, in order to reduce the TDD of the heteroepitaxy GaN film, some advances in nano-ELOG and the related technique of nano-pendeo epitaxial growth of GaN have been widely reported in the period circa 2005 to about 2013. Among them, some used the porous anodic alumina or porous SiO_2_ as growth mask and used the nano pore as the growth window^8^. While some directly grew GaN film GaN on porous GaN (nano-air-bridge structure), porous Si substrates or porous SiC substrates^9^,^10^,^11^. These nano-ELO methods are the development of the micro-ELO methods by reducing the size of the overgrowth region and the window. However, the high density nano nuclear windows produce a large amount of coalescence boundaries which increase the possibility of the appearance of the coalescence dislocation thus leads to the reduction of the total TDD to a limited range.

In this paper, we discuss a new ELO method, of which the overgrown regions are partly-contacted with underlying seed layer to suppress “wing tilts” and reduce coalescence threading dislocations (TDs). Figure 1 shows overgrowth diagram of the conventional ELO method, the partly-contacted ELO (PC-ELO) by conventional dielectric lithography film mask and the PC-ELO by the monolayer microsphere mask without lithography, respectively. The character of the PC-ELO method is that the growth mask has large growing-windows and small contact-windows, as shown in Fig. 1(c). The realization of the PC-ELO demand three key conditions: (i) the mask exists nano-scale contact-windows at the overgrowth regions; (ii) the suppression of material growth at the contact-window regions; (iii) the partly-contact of overgrowth layer material with underlying seed layer at the contact-windows. In the conventional ELO method, overgrowth regions either fully contact with a dielectric mask or non-contact with the seed layer using the “air-bridge” structure^12^,^13^. For the PC-ELO method, in order to contact with overgrown GaN, the seed layer remains exposed at the contact-windows. In this case, the growth at contact-windows must be suppressed to avoid nucleation at the contact-windows. The overgrowth layer fills the contact-windows and then contacts with the seed layer. It is only a small part of the overgrowth region for the contacting part with the seed layer. For the most parts, the overgrowth region and the seed layer are separated by mask. The contact-regions only accounts for a small part of the whole overgrowth region. Most region of the overgrowth layer is still separated with seed layer by mask. Therefore, the overgrowth layer (wing region) is partly-contacted with underlying seed layer. The PC-ELO method potentially eliminates the “wing tilt” in conventional ELO technique. Furthermore, considering microspheres could be used as the mask structure, we regard the close-packed monolayer microsphere as a PC-ELO mask method without lithography. The close-packed monolayer silica nanospheres or microspheres have been used to filter the TDs of GaN epitaxy layer on Si substrate and the GaN template^14^,^15^ and shown to have notable results in filtering the TDs. Some reported to use the partly-coated or selectively-placed nanospheres as the TDs filtering^16^,^17^. However, the microsphere masks reported before have distinctly different arrangement structure from the microspheres mask in this study. They could not realize the PC-ELO due to the lack of the suitable growth-window and contact-window. Figure 1 (3) shows the structure diagram of the monolayer microsphere mask in this study.

Herein, GaN growth was carried out using low-pressure metalorganic chemical vapor deposition (LP-MOCVD). A 1.2 μm thick GaN “seed” (base) layer was grown on 2 in. diam c-plane sapphire substrate, followed with a 500-nm-thick polystyrene (PS) layer fabricated by spin-coating method. Then the template is treated with oxygen plasma to improve the hydrophily of PS surface. Subsequently, silica microspheres were spin-coated on the PS, which were hexagonal closed-packed monolayer of sphere with a diameter of 1000 nm^18^. To remove PS layer and rearrange the microspheres, this step was followed by in-situ annealing above 600 °C in NH_3_ and H_2_ gases by MOCVD. During annealing, the temperature rose to 600 °C in 3 min and then was kept constant for 5 min. The regrowth was performed at a surface temperature of approximately 1000 °C. When the nucleation stripes nearly coalesced, the growth conditions were changed to a higher temperature (1030 °C) to courage rapid coalescence. Subsequent to coalescence, an additional 1 μm of planar GaN was grown with unchanged growth conditions.

Uncoated samples were characterized in cross section by scanning electron microscopy (SEM) using a Quanta 400 FEG field-emission microscope operating at 10 kV. Cathodoluminescence (CL) mapping was obtained using Gatan MonoCL3+ at 10 kV. Specimens for transmission electron microscopy were prepared by wedge polishing followed by standard Ar1-ion milling. Images were recorded on a Tecnai G2 F20 S-TWIN microscope operated at 200 kV. The surface topography was measured in tapping mode using an Agilent 5500 atomic force microscope (AFM). X-ray rocking curves of the GaN (0002) peak were recorded using Ge (220)-monochromated Cu Ka radiation in a four-circle diffractometer operating in receiving slit mode, with a 1.0 mm slit on the detector arm. The scattering plane was oriented [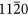] to measure the stripes perpendicular to the plane directions, so that the rotation (rocking) axis was parallel to the stripes along 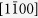 direction.

During annealing, PS interlayer melt as the temperature increased which caused the microspheres able to move freely in local area. Before the movement, the nearly symmetrically-arranged microspheres suffered attractive capillary force and were kept at high-energy equilibrium state. But this state was not stable. The symmetry was spontaneously broken after the movement originated from the imperfects, such as point defects or line defects. After annealing, it was observed that microspheres formed a multitude of close-packed regions in small scale, called “cluster”, which was used as the mask of overgrowth region. And the interspaces between the clusters were called “cluster gaps” as growing-windows. Particularly, the possible included angles of adjacent growing-windows in c-plane are 0°, 60° and 120°, which are corresponding to a crystallographic direction family of wurtzite GaN.

For 1000 nm diam. microspheres, the typical width of growing-windows is 700 ± 200 nm. A single cluster consists of approximately 25 close-packed microspheres. In other words, the distance between two windows is approximately 4 μm. In Fig. 2(c) the cross-section SEM micrograph shows the triangular-ridge nucleation stripes^12^. The surfaces of nucleation stripes are (0002) and 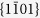 planes. Remarkably, even though there are interspaces (contact-windows) among the microspheres, the growth at the close-packed microsphere regions was suppressed. It is one of the key conditions to achieve the PC-ELO.

The cross-section SEM image in Fig. 2(c) also shows suppressed GaN growth at contact-windows region marked by the black arrows. GaN growth at contact-windows was suppressed at the height of microsphere center. During growing, the gas sources did not arrive at the seed layer surface for material growth, since contact-windows have a small width of approximately 150 nm and the large height/width value of approximately 6.7. In this case, the source used for growth only comes from the decomposition of seed layer GaN, especially, at the dislocation regions^19^. The SEM image also shows a large amount of decomposition pits. When the height of regrown GaN at contact-windows was equal to the height of microsphere center, regrown GaN completely filled the lower part of the contact-windows. The seed layer was not exposed, and therefore, the decomposition stopped. In consequence, the growth also stopped due to the lack of gas sources.

To show the contact condition between overgrowth layer and seed layer, Fig. 3 shows cross-section SEM micrograph of the nucleation stripe. Essentially, the sample structure looks like a “porous bridge”. The overgrowth layer and seed layer are “bridge deck” and “foundation base”, respectively. The overgrowth layer contacts with seed layer by the GaN in the intervals among microspheres functioning as the “piers” of porous bridge. Moreover, wing tilt would lead to the crystal direction mismatch between overgrowth layer and seed layer. The crystal direction mismatch results in the formation of defects at the contact interface which locates at the middle of pier. Therefore, the contact interface quality can be used to testify the existence of the crystal orientation mismatch. The defects at contact interface were characterized by TEM measurement. The cross-section TEM dark-field images in Fig. 4 showed that crystal quality at the contact interface. The white arrows mark the location of the contact interface. As a result, Fig. 4(b) with g = [0002] showed no screw dislocation and partial dislocation at the contact interfaces. Figure 4(c) with g = 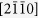 showed no edge dislocation and mixed dislocation at the contact interfaces. But a few stacking faults (SFs) were observed at the contact interface away from the window region in the Fig. 4(d) with g = 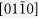. This might be explained by the fact that crystal mismatch begin to take from to some extent in the region away from the window region. In general, it has a high quality contact interface except a few SFs exist at partial interfaces. It is indirectly proved that the overgrown layer GaN continues the well crystal orientation of the seed layer by piers.

The rocking curve of the uncoalesced stripes and two representitive results with and without mask by rotating axis of 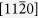 were also showed in Fig. 5(a). Peak splitting can be observed in previous studies to decrease wing tilt with or without mask. What is remarkable, though, is that uncoalesced stripes peak splitting due to tilt in this research is no longer detectable. Therefore, the wing tilt of nucleation stripes was completely eliminated by PC-ELO method. The tilt angle of 0.04° estimated by rocking curve is distinctly small compared to wing tilt angles for stripes by other high quality coalescence front method, which are larger than 0.11° with mask^6^,^20^,^21^,^22^ and 0.08^o^ without mask^13^,^23^. Moreover, Fig. 5(b) shows the rocking curve of the uncoalesced stripes and coalesced stripes by rotating axis of 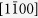 and 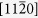. The single peaks of uncoalesced stripes have the full width at half maximum (FWHM) of 303 arcsec and 311 arcsec by rotating axis of 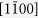 and 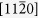, respectively. The single peaks of coalesced stripes have the FWHM of 216 arcsec and 206 arcsec by rotating axis of 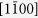 and 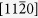, respectively. The uncoalesced stripes show a well crystal orientation, since the FWHM of the rocking curve by rotating axis of 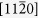 is small and approximately equal to that of the rocking curve by rotating axis of 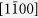. Moreover, the FWHM of the rocking curve from the uncoalesced stripes is only slightly larger than that of coalesced stripes. For conventional ELO with parallel stripe mask, the wing tilt is formed due to strain between overgrown GaN and dielectric mask^20^. Herein, the elimination of wing tilt could be explained as follows: on the one hand, there would not exist distinct strain between overgrown GaN and microsphere mask since every microsphere was spatially-separated with each other and substrate; on the other hand, the wing region was partly-connected with seed layer throughout regrowing progress, which had a supportive effect to wing GaN.

In order to specifically illustrate the TDs at the coalescence boundary, the CL mapping and AFM of the sample was given in Figs 6 and 7. TDs, as non-radioactive recombination center, were visualized as black dots in the CL mapping. Coalescence boundary was formed by the combination of two nucleation stripes with different included angle. Three coalescence boundary types A, B and C corresponded with the included angle 0°, 60° and 120°. The small CL mapping in Fig. 6 shows the distribution of the TDs in local area, of which the black arrow marks the worst coalescence boundary with high linear TDD. The worst coalescence boundary is the A-type coalescence boundary. AFM was performed in a 26 × 13 μm^2^ scan over an area covering more than one cluster (pattern period), as shown in Fig. 7. In the figure, there are several rows of tips and step-edge terminations step marked by white dashed line ellipses. They corresponded with the TDs at the worst coalescence boundary of the CL mapping in Fig. 6 due to the coincident arrangement and density. These TDs exists screw-component, since the tips and step-edge terminations in AFM image are the screw or mixed TDs^24^,^25^. In other words, in the vicinity of A-type coalescence boundary, there is existence of screw-component TDs and absence of pure edge TDs. In vicinity of the B- and C-type coalescence boundary, there is existence of pure edge TDs and absence of screw-component TDs. Due to the low misorientation of the overgrowth regions (wing), surface atom steps show rare discontinuities as they cross the coalescence front^6^.

An assessment of the TDs reduction can be directly made by counting the number of TDs in CL mapping^26^,^27^. The TDD reduces from ~2 × 10^9^ cm^−2^ to 6.2 × 10^7^ cm^−2^, which is about 30 magnitudes lower than that of seed layer GaN. It is much lower than that (4 × 10^8^ cm^−2^) reported by Li *et al.*^15^ using silica microsphere of 1000 nm in diameter as growth mask on GaN template. The screw or mixed TDD was estimated to be 1.8 × 10^7^ cm^−2^ by the tips and step-edge terminations in AFM image. The small CL image in Fig. 6 shows the distribution of TDs with low linear TDD, of which the worst coalescence boundary has the maximum linear TDD of 2 × 10^4^ cm^−1^. And the average linear TDD at coalescence boundary is approximately 6.4 × 10^3^ cm^−1^ calculated by dividing area TDD by coalescence boundary length per area. Such obvious reduction of TDs was not only due to the absence of the TDs threading at nucleation windows, which will be discussed in other paper, but also due to the depression of the nucleation tilt.

In conclusion, we demonstrate the PC-ELO method and achieve it without lithography by the monolayer microspheres mask. Realization of PC-ELO demands three key conditions. The monolayer microsphere mask was proved to meet these conditions. The coalesced front at the boundary of two nucleation strips has the average linear TDD of 6.4 × 10^3^ cm^−1^. The wing tilt was completely eliminated by partly-contacting with seed layer. As a development, PCELO method is also applicable to other crystal orientation GaN materials and other III-group nitride materials and their alloy materials.

## Additional Information

**How to cite this article**: Xiao, M. *et al.* A partly-contacted epitaxial lateral overgrowth method applied to GaN material. *Sci. Rep.* **6**, 23842; doi: 10.1038/srep23842 (2016).

## Figures and Tables

**Figure 1:**
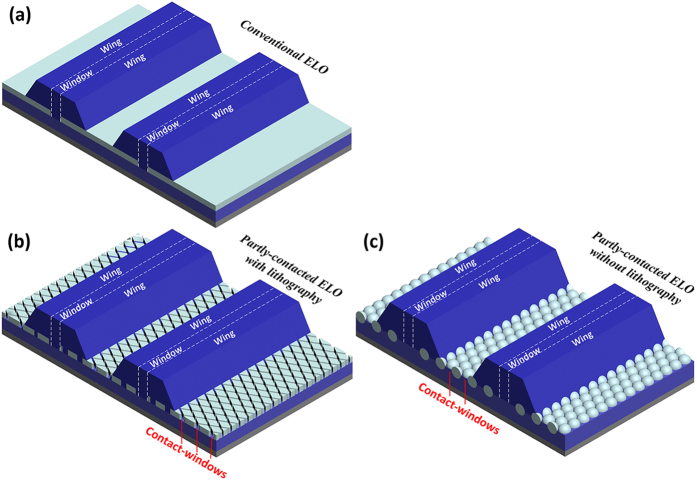
(**a**) Conventional ELO method and (**b**) PC-ELO method using film mask by lithography and (**c**) PC-ELO method using monolayer microsphere mask without lithography.

**Figure 2:**
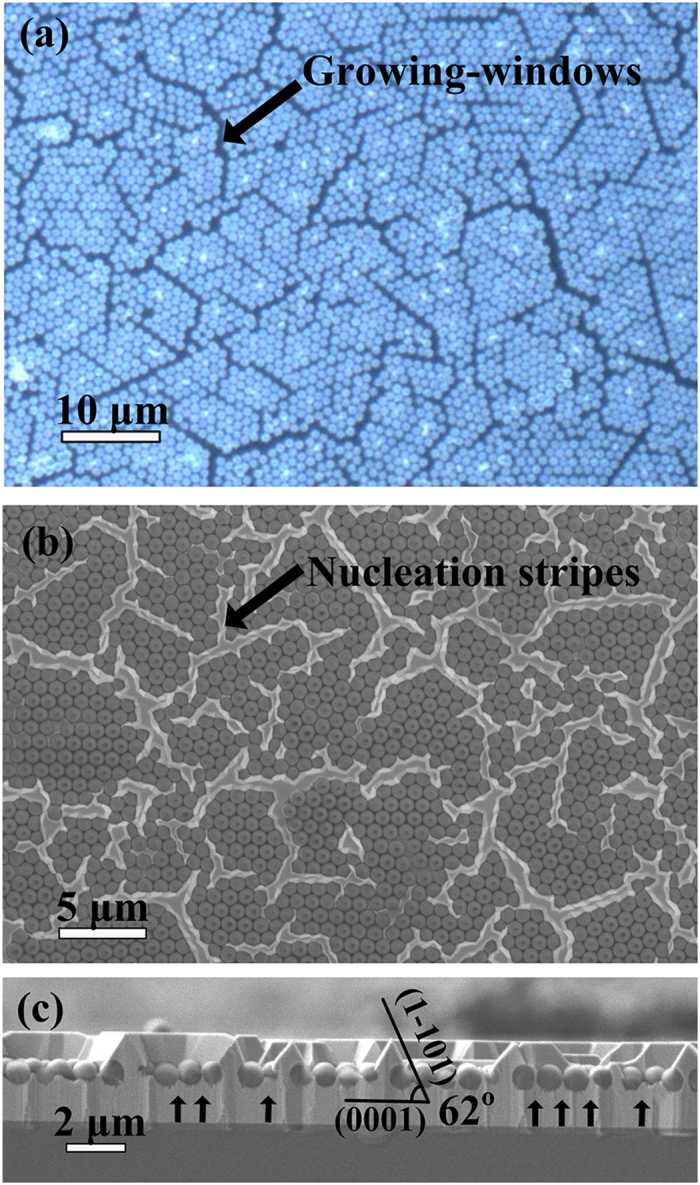
(**a**) The micrography of monolayer microspheres mask after annealing. (**b**) The surface and (**c**) cross-section SEM micrograph of nucleation islands. The GaN growth starts from the growing-windows marked by black arrow in (**a**). The growth at interval of microspheres was suppressed. The black arrows in (**c**) mark the impressed GaN growth at the microsphere intervals (contact-windows).

**Figure 3:**
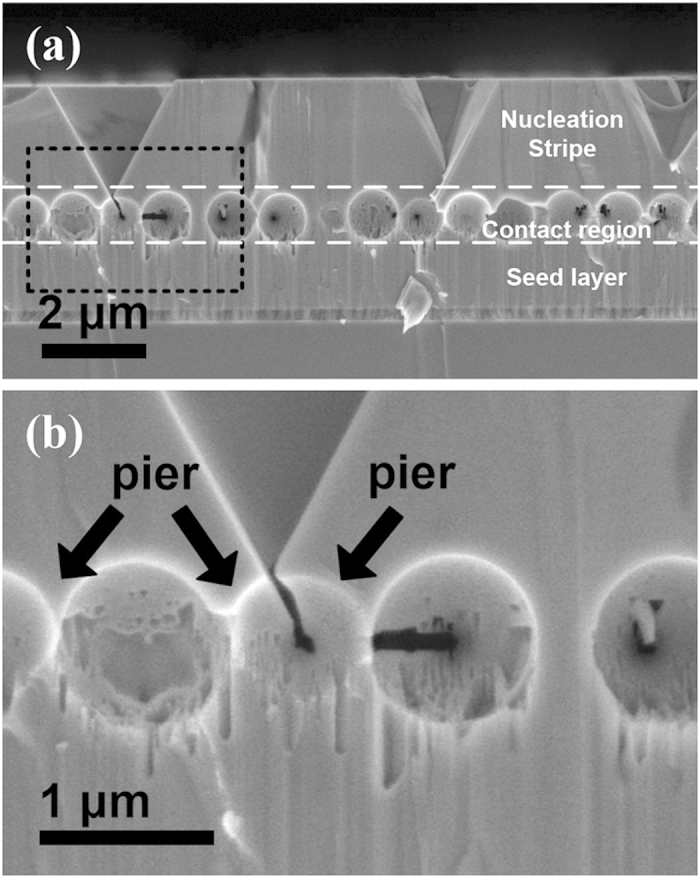
The cross-section SEM image of the nucleation stripe after removing microspheres. It shows that the overgrowth layer contacts with underlying seed layer GaN by “piers”. The black arrows mark the “piers”.

**Figure 4:**
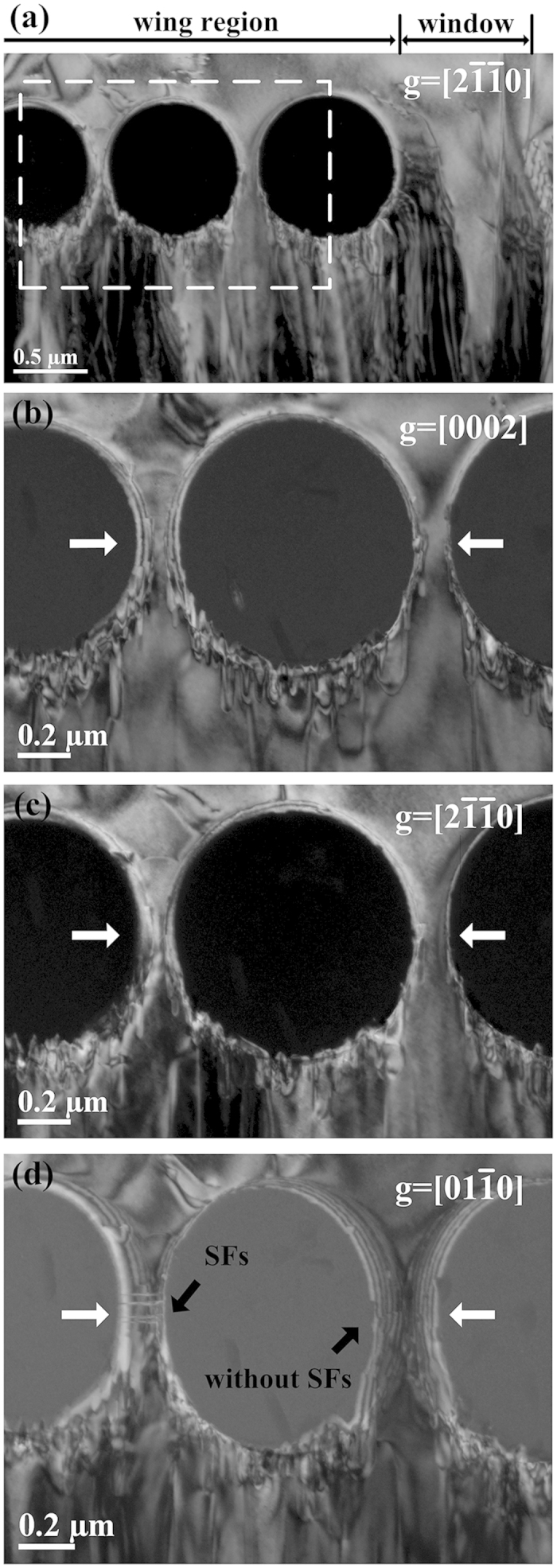
The contact interface quality analysis of overgrowth layer and seed layer by dark field TEM cross-section image with 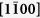 zone axis. (**a**) Dark field TEM cross-section image of the GaN epitaxial layer when g = 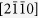. (**b–d**) correspond to the white rectangular zone in figure (**a**) with g = [0002], 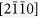 and 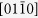, respectively. (**b**) g = [0002] highlights the presence of screw dislocations and partial dislocations that laterally terminate the stacking faults. (**c**) g = 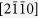 shows the presence of edge dislocations and mixed dislocations. (**d**) g = 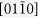 gives a clear contrast for the stacking faults. The contact interfaces are marked in figure (**b–d**) by the white arrows.

**Figure 5:**
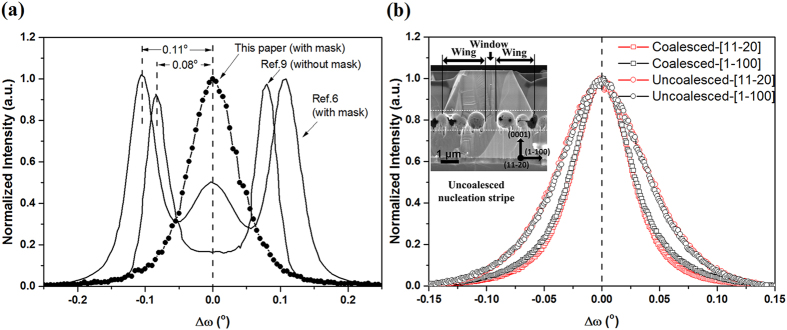
(**a**) Double crystal x-ray rocking curves of the GaN (0002) peak for uncoalesced stripes and two representive splitting peak rocking curves due to wing tilt with mask and without mask, measured with the scattering plane perpendicular to the 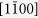 direction. (**b**) Double crystal x-ray rocking curves of the GaN (0002) peak for coalesced and uncoalesced stripes, measured with the scattering plane perpendicular to the 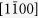 and 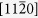 directions, respectively. The small cross-section SEM image in (**b**) shows the uncoalesced nucleation stripe.

**Figure 6:**
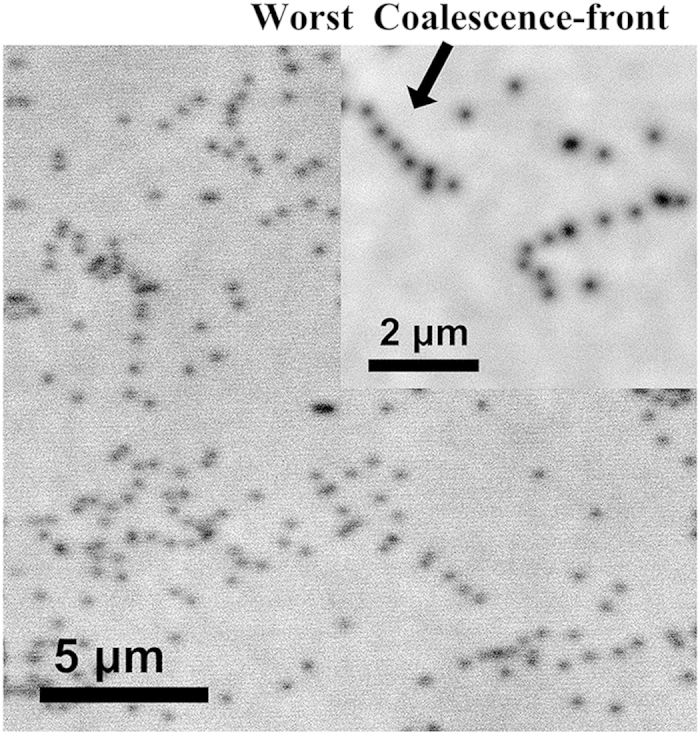
Large area CL mapping illustrates the low TDD of overgrowth layer. The small CL mapping at the upper-right corner shows the distribution of TDs at local, and marks the worst coalescence front by an arrow.

**Figure 7:**
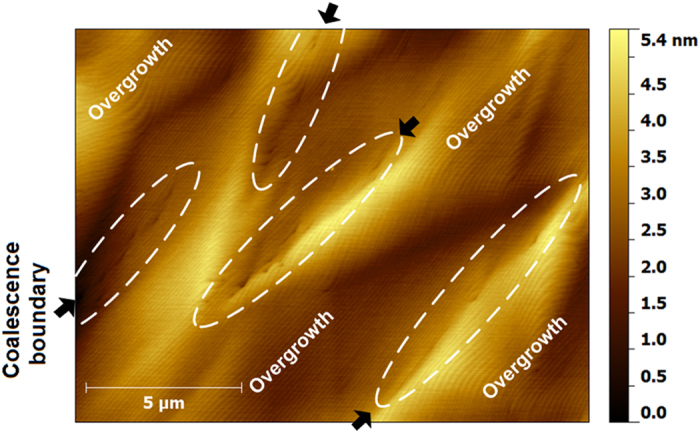
AFM micrograph of more than one pattern period, showing the coalescence fronts mark by dashed line ellipses.
